# LytU-SH3b fusion protein as a novel and efficient enzybiotic against methicillin-resistant *Staphylococcus aureus*

**Published:** 2019-12

**Authors:** Mortaza Taheri-Anganeh, Seyyed Hossein Khatami, Zeinab Jamali, Ahmad Movahedpour, Younes Ghasemi, Amir Savardashtaki, Zohreh Mostafavi-Pour

**Affiliations:** 1 Department of Medical Biotechnology, School of Advanced Medical Sciences and Technologies, Shiraz University of Medical Sciences, Shiraz, Iran; 2 Recombinant Protein Laboratory, Department of Biochemistry, School of Medicine, Shiraz University of Medical Sciences, Shiraz, Iran; 3 Cardiovascular Research Center, Shiraz University of Medical Sciences, Shiraz, Iran; 4 Student Research Committee, Shiraz University of Medical Sciences, Shiraz, Iran; 5 Department of Pharmaceutical Biotechnology, School of Pharmacy, Shiraz University of Medical Sciences, Shiraz, Iran; 6 Pharmaceutical Sciences Research Center, Shiraz University of Medical Sciences, Shiraz, Iran; 7 Maternal-Fetal Medicine Research Center, Shiraz University of Medical Sciences, Shiraz, Iran

**Keywords:** Staphylococcus aureus, Autolysin, LytU, SH3b, Enzybiotics

## Abstract

Methicillin-resistant *Staphylococcus aureus* (MRSA) is a challenging infectious agent worldwide. The ever growing antibiotic resistance has made the researchers to look for new anti-staphylococcal agents. Autolysins are staphylococcal enzymes that lyse bacterial cell wall for cell division. Autolysins can be used as novel enzybiotics (enzymes have antibiotic effects) for staphylococcal infections. LytU is a newly explored autolysin. SH3b is a potent cell wall binding domain that can be fused to lytic enzymes to increase their activity. The aim of this study was to design a novel and efficient fusion enzybiotic that could lyse staphylococcal cell wall peptidoglycan by disrupting the bacteria. LytU-SH3b fusion construct was synthesized and LytU was amplified through the construct, using overhang PCR. The fusion and native forms that had his-tag were synthesized by recombinant technology in *Escherichia coli *BL21 (DE3) strain and purified utilizing Ni-NTA agarose beads. LytU and LytU-SH3b activity and potency were assessed using plate lysis assay, turbidity reduction assay and minimal inhibitory concentration (MIC) tests. All these tests showed that LytU-SH3b has more activity and potency than LytU. LytU-SH3b has MIC 421 fold lesser than LytU. Finally, LytU-SH3b is a novel and efficient recombinant enzybiotic that can lyse MRSA as an alternative to chemical small molecule antibiotics.

## INTRODUCTION

*Staphylococcus aureus* (*S. aureus*) is a life-treating pathogen that can cause a wide variety of infections such as several connective tissues and blood infections [1]. On the other hand, the drug-resistant bacterial strains have become a global concern, especially in hospital environments, and by threatening the health of livestock [2]. The development and fast transmission of antibiotic-resistant *S. aureus *strains has generated a significant challenge for health organizations that has led to a search for alternative antibacterial factors. 

Recently, the wide spread of Methicillin-resistant *Staphylococcus aureus* (MRSA) infections, especially hospital-acquired ones has led to recruitment of stronger treatment approaches [3]. The large-scale use of some anti-staphylococcal drugs that inhibit the synthesis of *S. aureus* cell wall composed of peptidoglycan (PG) layers, create selective pressure to enhance the number of peptidoglycan (PG) layers [4]. Hence, lysis of PG with bacteriolytic enzymes serves as an appealing alternative in comparison to conventional small molecule antibiotics [5]. These lytic enzymes can be grouped into several categories according to their origin, called autolysins, exolysins and endolysins. They have the ability to lyse the cells if applied externally; hence, they can be considered as suitable antimicrobial agents [6].

Lysostaphin family are zinc-dependent endopeptidases targeting pentaglycine bridges of PG for degrading cell walls. They either play a role as an autolysin or a defense mechanism against competing strains [7]. Several investigations using lysostaphin as a bactericidal protein have produced promising results [8-12]. 

LytU is a recently explored member, belonging to lysostaphin family that can be introduced as a potential antimicrobial factor for *S. aureus* infections, since it degrades cell wall. The enzymatic activity of LytU is dependent on a unique Ile/Lys amino acids at position 151 in a loop close to the catalytic site. The effect of LytU on *S.* *aureus* cells was studied, using purified recombinant product, and according to the results it can effectively lyse *S. aureus* cells [7].

Lysins usually contain multi-domain construct, consisting of distinct catalytic and cell wall binding domains (CWBDs). The CWBDs improve the localization of lysins to the bacterial outer surface [13]. SH3b domain is known as one of the earliest CWBDs [14], but the role of SH3 domains is not completely understood, yet. They might act in some probable pathways including improvement of proteins localization, modifying their subcellular position and increasing the chance of accumulation of large multi-protein complexes [15].

The lysostaphin SH3b domain targets peptide bridges of the *S. aureus* PGNs [16]; therefore, conjugation of this binding domain with an enzymatic domain of *S. aureus* autolysin might result in amplified efficacy of lysostaphin in clinical treatment [3, 6]. In this regard, several recent fusion lysins with SH3b domains have had some improved qualities such as affinity or specificity [17, 18].

Today the recombinant technology is an inexpensive and efficient approach for protein synthesis [19]. *Escherichia coli (E.coli)* is the best known host for recombinant protein synthesis due to its high rate of growth, low feeding cost and known cellular physiology [20-22]. In this study, we created a recombinant fusion lysin protein, made of N-terminal enzymatically active domain of LytU and C- terminal SH3b domain. Finally, we compared the antibacterial effects of fusion and native LytU.

## MATERIALS AND METHODS

**Molecular cloning and expression: **LytU_26-192 _(167 amino acids) and SH3b (109 amino acids) amino acid sequences were obtained from NCBI Protein database (accession number: SA0205) and UniProt (accession number: P10547) [3, 7]. The sequences were back translated to nucleotide sequences using EMBOSS Backtranseq (https://www.ebi.ac.uk/Tools/st/emboss_ backtranseq/). The nucleotide sequence was optimized for expression in *E.coli* by GenScript (https://www.genscript.com). LytU-SH3b optimized construct was synthesized and sub-cloned in the NcoI and XhoI sites of vector pET28a by Biomatik Company (Biomatik, Ontario, Canada). The construct was then transformed into competent *E. coli* strain DH5α (Novagen, Wisconsin, United States) and the bacteria were cultured on Luria-Bertani (LB) agar. The plasmids were extracted using GeneJET Plasmid Miniprep Kit (Thermo Fisher Scientific, Massachusetts, USA). The extracted plasmids were used for colony PCR by pET28a universal primers through Taq DNA Polymerase 2x Master Mix RED (Ampliqon, Odense, Denmark) and double digestion by NcoI and XhoI restriction enzymes (Thermo Fisher Scientific, Massachusetts, USA). The confirmed plasmids were used for the next steps. Using PCR with overhang forward “ATG AGA ATT CTT ACA GAT CGT ACA GGC TAC G” primer and reverse “AAT TTT GTT TAA CTT TAA GAA GGA GAT ATA CCA TG” primer the active domain of LytU_26-192 _in addition to NcoI and EcoRI and restriction enzyme sites on 5ˊ and 3ˊ. The PCR was done using Phusion High-Fidelity DNA Polymerase (NEB, Massachusetts, USA) according to the kit protocol. PCR program included initiation denaturation at 95°C for 5 min, denaturation at 95°C for 30 s, annealing and extension at 72°C for 1 min (30 cycles) following final extension at 72°C for 10 min. PCR product was run on 1% agarose gel electrophoresis. This fragment was purified from agarose gel by QIAquick Extraction kit (Qiagen, Hilden, Germany) and ligated to the expression plasmid pET28a using T4 DNA ligase (Thermo Fisher Scientific, Massachusetts, USA). The result was two new recombinant vectors pET28a-LytU-SH3b and pET28a-LytU. Finally, both sequencing were performed using Bioneer (Daejeon, South Korea). Expression vectors were transformed into the *E.coli* strain BL21 (DE3) (Novagen, Wisconsin, USA). The bacteria were cultivated in LB medium and expression of proteins were induced by 1mM Isopropyl β-D-1-thiogalactopyranoside (IPTG) and performed for about 4 hours at 37°C. The induced bacteria were harvested using centrifugation 2150×g at 4°C.

**Purification:** *E.coli* strain BL21(DE3) cell pellet containing LytU-SH3b and LytU were suspended in lysis buffer (50 mM NaH_2_PO_4_, 10 mM imidazole, 300 mM NaCl, pH 8) and lysed by sonication for 6 times, each 10 s with 10 s intervals. Soluble material was harvested by centrifugation at 8000 ×g for 30 min, supernatant was incubated with Ni-NTA agarose beads (Qiagen, Hilden, Germany) for 2 h at 4°C. The mentioned sample was centrifuged at 1800 g at 4°C for 10 min in order to separate the flow-through. The beads were then washed twice with 5mL of washing buffer (50 mM NaH_2_PO_4_, 20 mM imidazole, 300 mM NaCl, pH 8). After washing, 1 mL of elution buffer (50 mM NaH_2_PO_4_, 250 mM imidazole, 300 mM NaCl, pH 8). The eluent was incubated at 4°C overnight and then centrifuged using the above protocol. 

The native and fusion protein size were predicted by ProtParam server (https://web.expasy. org/protparam/). Expression and purification accuracy was assessed by SDS-PAGE analysis in 18% (w/v) polyacrylamide gel containing 0.1% SDS. The native and fusion proteins samples were mixed with Laemmli 2X sample buffer (Merck, New Jersey, USA) and boiled for 10 minutes at 95°C on a hot plate. The SDS-PAGE was run at a voltage of 95 V. After an hour the gel was stained, using a Coomassie Blue solution for 15 minutes and de-stained overnight. For Western blotting, the run SDS-PAGE gel was transferred to nitrocellulose membranes (GE Healthcare, Illinois, USA) by a Mini Trans-blot Cell (Bio-Rad, California, USA) at a constant voltage of 200 V in a cold room overnight. The horseradish peroxidase conjugated anti-His antibodies were diluted at 1:1500 dilution, and used for the evaluation of recombinant proteins. The bound proteins were detected using diaminobenzidine (DAB) substrate (Sigma-Aldrich, Missouri, USA). The purified protein samples concentrates were defined by utilizing BCA^TM^ Protein Assay Kit (Thermo Fisher Scientific, Massachusetts, USA).

**Plate lysis assay: **Purified enzymes were serially diluted in saline lysis buffer (SLB;150 mM NaCl 10 mM Tris buffer, pH 7.5) to achieve 1000, 100 and 10 pmol/10 µl concentration. 10 µl of each dilution was spotted onto the plate of *S. aureus* strain SA113 (ATCC 3556) by adding to blank antibiogram discs. After, air dried Müller-Hinton agar plate was incubated at 37°C overnight. The control plate was spotted by a disc with no recombinant enzyme, which contained only the buffer. After that plates were compared with each other. 

**Turbidity reduction assays: **After *S. aureus* strain SA113 reached logarithmic phase (OD_600nm_= 0.4-0.6) in tryptic soy broth (TSB) medium at 37°C, they were collected using centrifugation at 4°C. They were maintained in a buffer containing NaCl 150mM, Tris-Cl 10mM pH=7.5 on ice for 4 hours. One hundred µl of enzymatic buffer containing 5 µg/ml (LytU or LytU-SH3b) was added to each 96 well plate. The dilution of the recombinant enzybiotics was performed in cation-adjusted Mueller Hinton broth (Becton Dickinson, New Jersey, USA) supplemented with 2% NaCl (Merck, New Jersey, USA) and 0.1% bovine serum albumin (BSA) (Sigma-Aldrich, Missouri, USA). MIC determinations were performed in the presence of 0.1% BSA to inhibit non-specific recombinant enzybiotics binding to the polystyrene plate. Reactions were initiated by adding 100µl of *S. aureus* cells together. The control reaction was done without any enzyme and containing only bacteria and buffer. Every 2 min in 30 min absorption was read in 600 nm. The maximum rate for each was reported as turbidity reduction rate. 

**Minimal inhibitory concentration (MIC) determination** **: **One hundred µl of TSB medium containing 40000 CFU/ml *S. aureus* strain SA113 was harvested in 96-well plate. 50 µl of the native LytU and the LytU-SH3b enzymes (with concentration ranging 0.25-0.00025 mg/ml) were added to each well, a control well without any enzyme was considered in each assay. Plates were incubated in 37°C with shaking at 150 rpm for 24 hours. Absorption of each well were measured in 600nm and the minimal inhibitory concentration was determined according to wells without any absorption.

**Statistical analysis: **The differences in LytU and LytU-SH3b disc diffusion diameters and MIC values were verified using SPSS software (version 22). The t-test was used for data analysis. All tests were performed 3 times and P<0.05. 

## RESULTS

In this study, the LytU sequence without 25 first signal peptide amino acids were fused with SH3b domain of *Staphylococcus simulans* lysostaphin. The fusion construct was synthesized and sub-cloned into pET28a by the aforementioned company. Using the fusion construct as the template, the sequence of native LytU, was amplified using overhang PCR and was sub-cloned into pET28a (The product size was 553bp).

**Figure 1:**
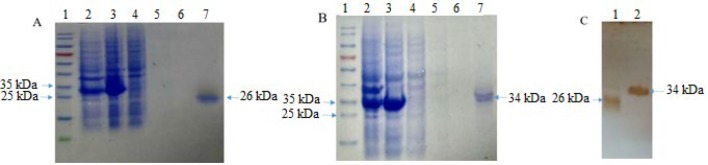
**SDS-PAGE and Western blotting analysis of LytU and LytU-SH3b**. A: line 1 protein size marker, Line 2 and 3, uninduced and induced expression by IPTG, line 4 prewash, line 5 and 6 consecutive washes, line 7 final elution of LytU (26 kDa). B: line 1 protein size marker, Line 2 and 3, uninduced and induced expression by IPTG, line 4 prewash, line 5 and 6 consecutive washes, line 7 final elution of LytU-SH3b (34kDa). C: line 1 LytU, line 2 LytU-SH3b.

Colony PCR, restriction enzymes were double digested, and the sequencing confirmed the correct direct and sequences of the constructs. Expression of the constructs under control of T7 promoter were induced using IPTG. The sizes of LytU and LytU-SH3b were predicted about 24 and 32 kDa, respectively. SDS-PAGE showed that both were soluble and suspended in bacterial supernatant. SDS-PAGE showed that they were in eluent solution, and they were no pollutant band along. Western blotting confirmed SDS-PAGE results (Fig. 1). Thus, the recombinant proteins purification was perfect. The lytic activity of LytU and LytU-SH3b were assessed using some antimicrobial tests. In plate lysis assay, diffusion of the recombinant enzybiotics were assayed. The diameter of not grown regions on *S. aureus* strain SA113 cultured plates were investigated using 1000, 100, 10 pmol/10 µl concentrations of the native and fusion proteins. 

**Figure 2:**
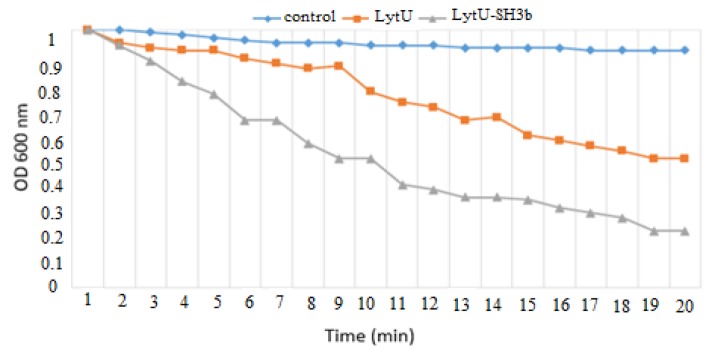
Turbidity reduction assay

The plate lysis zone measurement results showed that in same concentrations, the growth inhibition zone diameter of LytU-SH3b was more than LytU, as the fusion protein had more disc diffusion and lytic activity. The differences were significant (P<0.05) (Table 1). These results were confirmed by turbidity reduction assays. The turbidity assay measures the reduction in optical density (OD) of a suspension of *S. aureus* when exposed to the enzybiotics. The time to decrease to half of the starting OD (OD_50_) with 5 µg/ml concentrations of the native and fusion protein were determined, and the time for both proteins were calculated (Fig. 2). The OD_50_ time for LytU and LytU-SH3b were determined around 19 and 8.5 minutes, respectively. The result showed LytU-SH3b had more lytic potency by this assay. 

**Table 1:** Disc diffusion diameter for various concentrations of LytU and LytU-SH3b

**Assay**	**Type of protein**	**Concentrations (pmol/10 µl)**		
		**1000**	**100**	**10**
Disc diffusion diameter (mm)	LytU	18.2±2	3.7±1	1.8±0.5
	LytU-SH3b	25.2±3	7.8±1.5	4.0±0.3

The MIC of the recombinant native and fusion proteins for the methicillin-resistant *S. aureus,* which resulted in no detectable bacterial growth were determined. The MIC for LytU-SH3b was 421 fold lesser than another one. The MIC differences were significant (P<0.05) (Table 2). This means that LytU-SH3b lytic activity and potency was much more than LytU. 

**Table 2:** Comparison of LytU and LytU-SH3b MICs and some small molecule antibiotics

**Antimicrobial agents**	**Minimum inhibitory Concentration (MIC)**		**References**
	μg mL^−1^	NM	
LytU	97±4	4040±166.67	-
LytU-SH3b	0.23±0.08	7.19±2.5	-
Rifampin	0.02	24	[27]
Clindamycin	0.1	240	[28]
Vancomycin	1.4	970	[29]
Daptomycin	25	15000	[27]

## DISCUSSION

Methicillin-resistant *S. aureus* (MRSA) is the reason for several difficult-to treat infections in humans. The first medicine against MRSA, was methicillin, but due to its side effects, methicillin is no longer clinically used. Furthermore, *S. aureus* is a multi-resistance pathogen that has resistance ability even for currently used and future antibiotics [23], due to their ability to lyse the peptidoglycan of cell walls. Several bacteriolytic enzymes can be suitable alternatives for the common antibiotics. These enzymes are grouped into four-well-studied categories including autolysins [3]. LytU is a novel autolysin that was discovered recently, belonging to *S. aureus* [7]. The structure of most active lysins is comprised of several regions including catalytic and cell wall binding domains (CWBDs). The role of CWBDs is to anchor the enzyme to the peptidoglycans of cell surface and to facilitate the bacteriolytic activity of catalytic domains [3]. SH3b domains are some of the well-studied PG hydrolase CWBDs [6]. The lysostaphin SH3b domain is one of the first identified cell wall binding domains. It was shown that an essential 92 residues at the C-terminal of lysostaphin is crucial for specific binding to the cell walls according to their protein types [14]. The SH3b tertiary structure has a groove between β1 and β2 sheets which plays as a pentaglycine binding site. Steric exclusion of pentaglycine Cβ atoms directly enhance SH3b specificity. Therefore, major chain conformations of the above mentioned groove are specific for glycine, and are not accessible for the other amino acids [24]. The presence of some ions in the catalytic reaction medium might inhibit the enzymatic activity of lysostaphins catalytic domains, the SH3b can help to overcome this limitation [6].

Becker *et al.,* indicated that the fusion of a staphylococcal cell wall binding domain with a poor streptococcal lytic sequence can raise its staphylolytic activity [25]. Becker *et al.,* studied the phage Twort endolysin (PlyTW). It has three domains including amidase-2 domain, amidohydrolases/peptidase domain and SH3b domain. The results showed that in comparison to the complete protein, lack of CBD can cause the enzyme activity to be reduced to approximately 10X [17].

We hypothesized that fusing LytU to SH3b could expand its activity, potency and binding to PG of bacterial cell walls. Hence, we designed a fusion construct containing a LytU; a recently discovered autolysin and SH3b; an efficient CWBD. The recombinant native and fusion forms were expressed, purified and their antibacterial potency were compared.

Mao *et al.* fused the phage 187 endolysin (Ply187) endopeptidase domain with the LysK SH3b domain and showed the chimeric protein to be more potent than the native endopeptidase against *S. aureus *[26].

Osipovitch *et al.* created two fusions of LytM; a *S. aureus* autolysin. In these fusions the catalytic domain of LytM was in N-terminal and *S. simulans* lysostsaphin CWBD (SH3b) resided in C-terminal. The study results showed that their activity had increased significantly in comparison with the native LytM. One of the fusions that had no linker between the two segments, had anti-staphylococcal activity 540 fold more than the native form [3]. LytM and LytU belong to lysostaphin family. 185-316 amino acids of LytM and 47-182 amino acids of LytU are homologs and are M23 endopeptidase family members [7]. 

Hence, we designed a fusion construct that LytU fused to SH3b domain without any linker. In accordance with the above mentioned studies the results of our fusion protein had more effective anti- staphylococcal enzybiotic and had MIC 421 fold more than the native one. Our constructs MICs in comparison with some common small molecule anti- staphylococcal antibiotics are shown in Table 3. The MIC values for small molecule chemical antibiotics are in the range of μg mL^−1 ^to sub μg mL^−1^ values. Highly active antibiotic biomolecules like peptides have low MIC values, although the larger antibiotic peptides have higher potency based on molar basis [3]. In this study, LytU-SH3b showed sub-μg ml^−1^ MIC values, which means it had greater potency than small molecule antimicrobial agents and antibiotic peptides. 

In conclusion, LytU-SH3b is a novel and efficient fusion enzybiotic that harbors an enzymatic domain and cell wall binding domain. This enzybiotic can be a suitable alternative to traditional chemical antibiotics against drug resistant *S. aureus* strains.
